# HIV, 95‐95‐95 and the allocative efficiency fallacy: why treating everyone makes sense from a humanitarian, clinical, economic and disease control perspective

**DOI:** 10.1002/jia2.25191

**Published:** 2018-10-14

**Authors:** Reuben Granich, Somya Gupta, Brian G Williams

**Affiliations:** ^1^ Independent Public Health Consultant San Francisco CA USA; ^2^ Independent Public Health Consultant Delhi India; ^3^ South African Centre for Epidemiological Modelling and Analysis Stellenbosch South Africa

**Keywords:** HIV, AIDS, HIV treatment, treatment as prevention, epidemiologic model, resource needs

Dear Editor,

HIV remains a major public health threat with an estimated 37 million people living with HIV, 1.8 million annual infections and 940,000 deaths a year 1. Recent population‐based studies suggest a marked decline in HIV incidence and deaths largely due to HIV treatment expansion in countries in eastern and southern Africa and other settings 2. This decline calls into question the mainstream modelling framing and assumptions around treatment as prevention for illness, death and transmission. The recent article on the Optima models from 23 countries is a case in point as the paper is flawed in both its framing and its parameterization of the HIV response 3.

The Optima models 3 do not take into account the global 90‐90‐90 by 2020 or the 95‐95‐95 by 2030 target 4. The 90‐90‐90 target includes 81% of people living with HIV on antiretroviral therapy (ART) or 73% on treatment and virally suppressed 4. Although resources are limited, multiple studies and sources suggest that only around 50% to 60% of global resources for the HIV response are allocated to testing and treatment 5, 6, 7, 8. Despite this budget space, since Optima does not use 90‐90‐90 or 95‐95‐95 as a target, instead their models are limited to an average 15%–64% increase in budget allocations resulting in an average of only 42% ART coverage – far less than the 81% coverage envisioned by the 90‐90‐90 target. The models set allocation and treatment objectives far too low, implicitly denying treatment to the majority of people living with HIV, resulting in millions of avoidable infections, illnesses and deaths.

Optima is an “allocative” efficiency model; however, given the global 95‐95‐95 target and flat budget, the challenge for the world is technical efficiency – spending existing money in the best and most impactful way. There is an important dynamic between technical and allocative efficiency, however, and most existing models make poor spending assumptions – overpricing treatment and overvaluing other interventions. In contrast to many other prevention interventions, there have been major efficiency gains in delivering treatment and going forward it will become even less expensive. The lower cost coupled with the major clinical and public health impact means that in the future many prevention activities will not make economic sense. Put differently, if the Optima model does not capture the multiple prevention impacts of treatment, relative low cost and the systemic savings, the result is an erroneous allocation away from treatment.

Despite the problematic de‐prioritization of treatment, the models do conclude that increasing treatment coverage could lower incidence, a finding common among most models published over the past decade 7, 9, 10, 11, 12, 13, 14. However, in contrast to this finding, the Optima models allocate resources to “prevention” as equally or more important as providing access to treatment – ignoring the fact that treatment is the most powerful form of prevention. This renders the models inherently inefficient resulting in the unrealistic estimation of a 185% resource gap. Even if the other scientifically proven forms of prevention were the more cost‐effective, it would still be inhumane and unethical to strategically plan to not offer treatment to the majority of people living with HIV in the hopes that the other prevention interventions may prevent future infections.

In layperson's terms, the Optima models are framed and parameterized to result in the majority of people dying due to lack of access to treatment while prioritizing protecting others with non‐treatment prevention interventions. Prioritizing expanding access to treatment makes sense from compassionate, efficiency and disease control perspectives. By prioritizing and maximizing efficiencies, we have the resources to offer life‐saving treatment to achieve 95‐95‐95 or at least 86% of people living with being virally suppressed, a major milestone towards the end of AIDS. A secondary, but equally important, benefit of reaching everyone with treatment is that it is the most effective form of prevention. Simply put, where prevalence is high, resources should be prioritized to test everyone once or twice a year. For those who test positive provide immediate optimal treatment – for those who test negative but who are at significant risk, such as sex workers, men who have sex with men and people who inject drugs, provide other prevention interventions. As incidence comes down the programme will have resources to devote to active case management and outreach to sexual and needle‐sharing partners. Models used for national strategies should reflect the humanitarian, efficiency and disease control priority to expand access to treatment as the foundation for successful efforts to end the AIDS epidemic.

All the very best,

Reuben Granich

Somya Gupta

Brian Williams

## Competing interests

Authors have no competing interests.

## Authors’ contributions

RG, SG and BGW contributed equally to conceptualizing, developing arguments, producing and finalizing the final draft. All authors have read and approved the final manuscript.
